# From Real-world Individuals’ Data to National Health Indicators: Multiphase Pilot Study in Gabon

**DOI:** 10.2196/35176

**Published:** 2022-10-07

**Authors:** Aimé Patrice Koumamba, Edgard Brice Ngoungou, Jean Engohang-Ndong, Euloge Ibinga, Raymond Ondzigue Mbenga, Gayo Diallo

**Affiliations:** 1 Centre de recherche sur la santé des populations de Bordeaux Institut national de la santé et de la recherche médicale 1219 Université de Bordeaux Bordeaux France; 2 Research Unit in Epidemiology of Chronic Diseases and Environmental Health, University of Health Sciences Libreville Gabon; 3 Department of Biological Sciences, Kent State University at Tuscarawas Kent, OH United States; 4 Laboratoire d'Informatique fondamentale appliquée de Tours University of Tours Tours France

**Keywords:** health data quality, decision support information system, low- and middle-income countries, LMICs, mobile phone

## Abstract

**Background:**

Achieving health goals requires informed decision-making supported by transparent, reliable, and relevant health information. This helps decision makers, such as health managers, to better understand the functioning of their health system and improve their ability to respond quickly to health demands. To achieve this, the health system needs to be supported by a digitized decision-making information system. In Sub-Saharan African countries, inadequate digital infrastructure, including limited internet connectivity and insufficient access to appropriate computer software, makes it difficult to collect, process, and analyze data for health statistics. The processing of data is done manually in this case; however, this situation affects the quality of the health statistics produced and compromises the quality of health intervention choices in these countries.

**Objective:**

This study aimed to describe the conceptual approach of a data production and dissemination platform model proposed and implemented in Gabon. More precisely, it aimed to present the approach applied for the multidimensional analysis of the data production and dissemination process in the existing information system and present the results of an evaluation of the proposed model implemented in a real context.

**Methods:**

The research was carried out in 3 phases. First, a platform was designed and developed based on the examination of the various data production and indicator generation procedures. Then, the platform was implemented in chosen health facilities in Gabon. Finally, a platform evaluation was carried out with actual end users.

**Results:**

A total of 14 users with 12 years of average experience in health data management were interviewed. The results show that the use of the proposed model significantly improved the completeness, timeliness, and accuracy of data compared with the traditional system (93% vs 12%, *P*<.001; 96% vs 18%, *P*<.001; and 100% vs 18%, *P*<.001; respectively).

**Conclusions:**

The proposed model contributes significantly to the improvement of health data quality in Gabon.

## Introduction

### Background

**Figure 1:**
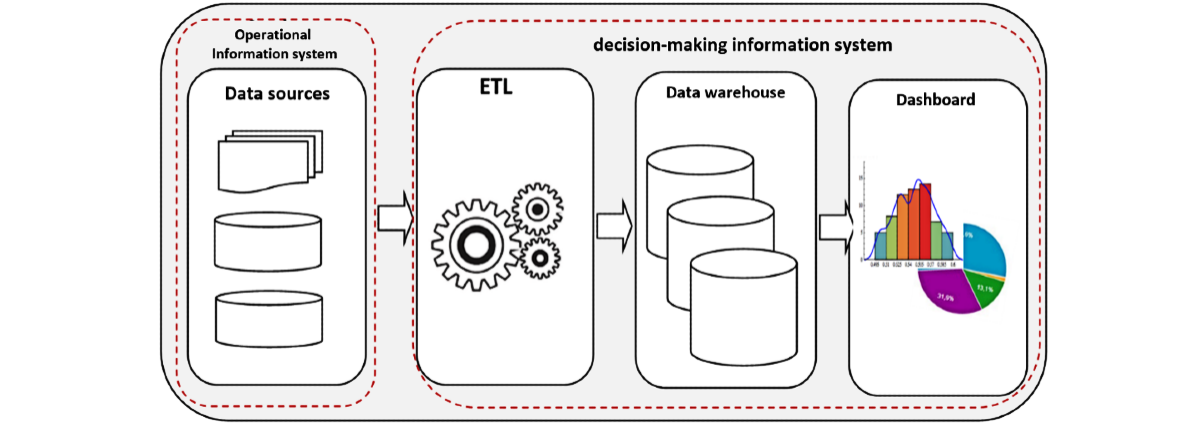
Simplified workflow of a data-enabled decision-making process. ETL: extraction, transformation, and loading.

In the 21st century, a reliable health information system (HIS) must rely on a digitalized decision-making information system (DMIS), which is “a bundle of subject-oriented, integrated, time-evolving, and nonvolatile data designed to aid managers in their decision-making” [1]. The performance of such a DMIS depends on its ability to manage data from heterogeneous sources, and it uses a data warehousing approach to help the HIS perform well. Thus, it is important to consider transactional information systems in the development of a DMIS (Figure 1).

In any organization, the quality of the choice of interventions to be undertaken is linked to the visibility that decision makers have of the performance of their organizations. For any health system, the decision-making level needs to rely on a DMIS, which, by means of an extraction, transformation, and loading (ETL) approach, can process data from the transactional systems [2]. This then allows the understanding that the performance of a DMIS in terms of producing quality data depends on its best interaction with transactional systems.

### Challenges

Owing to restricted digital infrastructures, including limited internet connectivity or insufficient access to proper computer software, it is difficult for many health systems to obtain high-quality data. According to the Global Health Observatory, 81 nations (42%) gather data of extremely low quality. This data quality issue is a significant hindrance to the management of health services, the creation of health statistics, and even the monitoring of health. In Sub-Saharan African (SSA) countries, the problem is even more significant and undermines the quality of choices for health interventions [3]. In these countries, the organization of HIS seems to have some shortcomings, which could explain the data quality issue [4].

### HIS Implementation in SSA Countries

The implementation of HISs in SSA has been largely supported by the recent version of District Health Information Software 2 (DHIS2; Health Information Systems Programme) [5]. The latter was developed under the leadership of the University of Oslo in Norway [6,7]. The management of health data through the DHIS2 platform is now effective in many SSA countries [8]. However, despite this, the performance of information systems remains problematic in many SSA countries, including in Gabon. Indeed, in most health facilities, aggregated data are collected manually by data managers [9], who are in charge of integrating these aggregated data into the DHIS2 platform, contextualized according to the country and health system. Nevertheless, this approach to manual upstream data processing often results in a large number of human errors, including incomplete data. Therefore, the manual data collection techniques are onerous and time-consuming and increase the workload of data managers who must collect data from the many health programs that generate field data [10]. Although DHIS2 is widely implemented in several SSA health systems, this platform does not appear to account for data production at the fundamental level, that is, at the health facilities where patients are seen by a health care provider (apart from the recent e-Tracker service in the context of the COVID-19 pandemic). However, because of the huge volume of data produced, their exploitation would be facilitated by the use of data ETL technologies to permit multidimensional analysis at the smallest feasible resolution if they were digitized. The poor use of digitalized hospital information systems is not only a source of demotivation for data managers but also a significant barrier to the development of trustworthy indicators for directing decision-making in health services. To rectify this predicament, some health system officers have decided to use the e-Tracker DHIS2 technology to digitize all data management tasks [7]. However, despite such initiatives to help improve the quality of data for decision support, the difficulties in getting integrated information systems that take into account individual data are still present. In most resource-limited countries, specifically in SSA, the implementation of decision support tools is largely supported by the DHIS2 system and its data warehouse (DW) platform. Unfortunately, this platform is not focused on data processing at the operational level, particularly at the level of primary data sources (consisting of transactional systems and where data are produced). However, the DHIS2 platform facilitates the collection of aggregated data at the level of the health department, health region, and central programs that are vertically oriented (HIV, tuberculosis, malaria, etc). The operational level such as hospitals, clinics, and health centers, which beyond the operational (medical care), which ensures the day-to-day management of the organization, is rarely (or even not) taken into account. The process of data processing, also known as ETL of data into the DW, is done manually in most cases [11].

Similar to many other SSA countries, Gabon has a data quality issue [12]. To make the issue even more complex, Gabon has a fragmented information system without any formal coordination. In a context in which a health system generates voluminous, very diverse, and heterogeneous data, the information system should be based on well-structured transactional information systems and be continually fed. In Gabon, unfortunately, upstream of the HIS, data processing at the operating system level is carried out manually. As a result, the data quality is poor [4]. Overall, the quality of the data produced may be affected by the lack of data completeness (ie, the data produced are incomplete), data accuracy (ie, data recorded are different from those actually produced), and data timeliness (ie, data are made available to decision makers after a long delay).

According to the above observations, the following question arises: what information system should be adopted to improve the performance of the system and provide quality data? In order to answer this question, a pilot study was undertaken in Gabon. The main purpose was to implement a platform integrating both health-related operational and decision-making data.

### Objectives

The main objective of this study was to describe the design of the conceptual approach of the platform and its implementation in Gabon. It enabled the multidimensional analysis of health information for management support to analyze the data production and dissemination processes in the existing information system and evaluate the multidimensional model and framework implemented in a real context setting.

### Gabon and Its Health System Context

**Figure 2:**
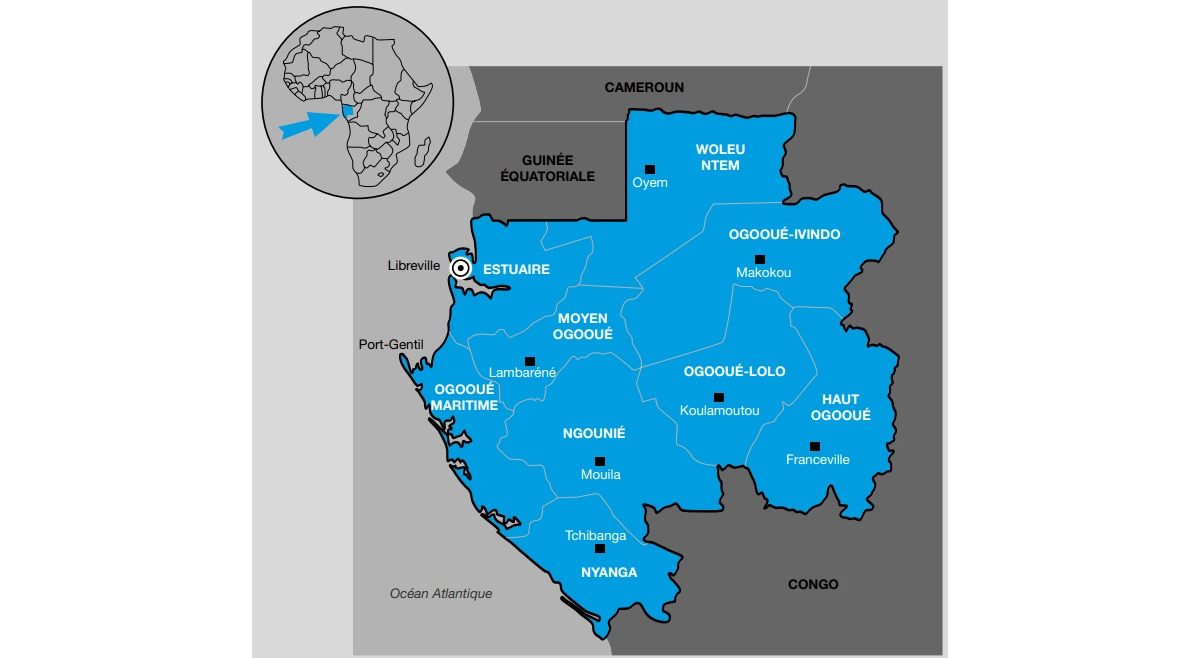
Geographical location of Gabon.

Located in Central Africa, Gabon is a country with a surface area of 267,667 km^2^ and an estimated population of 1,811,079 as of 2021. It is bordered by Equatorial Guinea, Cameroon, Congo Brazzaville, and the Atlantic Ocean, which extends for about 800 km along its coast (Figure 2) [13].

**Figure 3:**
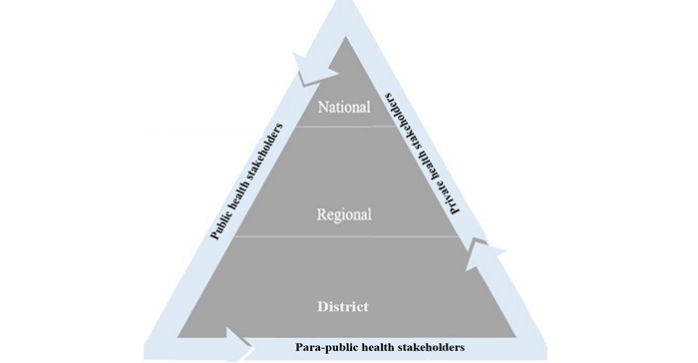
The health system’s stakeholders and pyramidal organization in Gabon.

The health system in Gabon comprises 3 types of stakeholders: public, private, and para-public. The entire system is structured as a pyramidal organization, with health districts at the bottom, regions in the middle, and the national level at the top, which coordinates and centralizes all health care activities and data in the country (Figure 3).

When the digital infrastructure is adequate, health care institutions are typically digitalized with a reliable internet connection, computers, software, and health care management software. Moreover, all patient care operations, including data-generating activities, are frequently combined in a digitized patient file. This is the primary data source used by ETL systems to automatically populate clinical DWs. However, in Gabon, this is not always the case. Thus, health facilities are compelled to collect data using paper records, which makes it challenging to use these data for decision-making [12].

Currently, the processes used in Gabon do not permit decision makers to differentiate between aggregated data collected from the district and individual health information data collected from each health facility inside the district. As an illustration, in 2019, the Komo-Mondah Health District, which consists of 162 health facilities, reported malaria as the leading cause of medical visits in the district. It accounted for 49% of all recorded health consultations (2966 cases out of 6054 medical consultations). However, investigators (statisticians) were unable to determine the distribution of malaria cases by health facility within the health district. This lack of detail does not promote targeted and relevant health management decision-making.

Therefore, it was necessary to brainstorm and create a system or model that would be suited for the automatic gathering and storage of health data and could link these data with their production sources. A system that could function effectively even in the absence of a dependable internet connection or limited access to computers. Such a system could enable the end user to enter data into the system using a smartphone connected to the national mobile network. This option is widely available in Gabon, as more than 80% of the mobile phone users have access to a smartphone with a reliable internet connection that enables the transfer of data in almost real time, thus eliminating the need to record health data on paper-based forms and allowing health professionals to instantly access the tool using previously assigned credentials.

The purpose was to minimize manual and delayed data processing, both of which are potential sources of errors that could influence data quality. The model suggested in this study includes built-in variables that provide the entry of health data contextualized by health facility and by health service, thereby enabling data processing employees and investigators to analyze digitized data with relevant details.

The Gabonese health system is organized into 10 health regions and 51 health districts. It comprises 1043 health facilities, the main ones being 3 university hospital centers, 9 regional hospital centers, 51 medical centers, and 2 large hospitals with a capacity comparable with that of a university hospital center [14].

## Methods

### Overview

The implementation of any HIS requires the consideration of all the processes that contribute to the production of data and the dissemination of information. This makes it possible to highlight the sequence of activities enabling the transformation of data into information, information into knowledge, and knowledge into action [14]. Thus, to define the different relationships between the data structures, in this study, data were first collected from semidirective interviews with the different health actors involved in these different processes. Following data collection, a model of DMIS based on 2 modeling methods was proposed. First, the process method aims to break down activities step by step to study their functioning and their interactions to improve the organization of the overall system. For this study, we used Bizagi, a tool for designing architecture models in information systems development [15]. This tool allowed the research team to describe and represent the different data collection processes in the Gabonese HIS. Second, the method of study and computer implementation for business systems is a popular method in French-speaking countries, whereby an information system is designed independently of the technical choices for its implementation [16]. This allowed us to design the conceptual data model while identifying the different actors and how they interacted with each other at different levels of the system.

### Analysis of the Different Interactions in the Data Production Process and Modeling of the DMIS

Owing to the fact that operational systems such as HIS feed DMIS with data, it was vital for the purposes of this study to analyze the numerous activities that occur within the health care facilities, where the primary data are produced. This allows for a realistic depiction of the many interactions among the actors involved in data production. This description emphasizes the sequence of medical care activities that generate diverse sorts of data. The operational level, which represents the numerous information sources, was now digitalized, and the data integration requirements were easily met by designing an ETL procedure. The latter ensures that the data are processed and loaded into a specific DW [17]. In cases where data production processes were not digitalized at the initial production level, it was determined that for the purposes of this pilot study specifically, which was focused on enabling decision support, epidemiological surveillance, and monitoring and evaluation of the performance of the health system (as opposed to medical follow-up or care), the framework should address all processes from data production to information dissemination.

### Data Production Process

The production of data that were fed into the DMIS was based on a set of activities that were associated with medical diagnoses performed in a given medical service (operational system). During the medical visit phase, the patient was identified as the central participant around whom the medical staff performed all activities associated with the medical consultation, which produced a set of data. These data consisted mainly, but not exclusively, of 3 types of records:

- *Sociodemographic data*, which were information related to age, gender, nationality, place of residence, and date of consultation of the patient. In addition, the records indicated the name and role of the health provider.
- *Economic data*, which were information associated with the health insurance status, occupation, and copayment rate of the patient. With regard to health insurance, it was systematically indicated whether the patient received health coverage from the government.
- *Clinical data*, which included vital signs, medical diagnoses, drugs prescribed, and types of laboratory tests prescribed.

**Figure 4:**
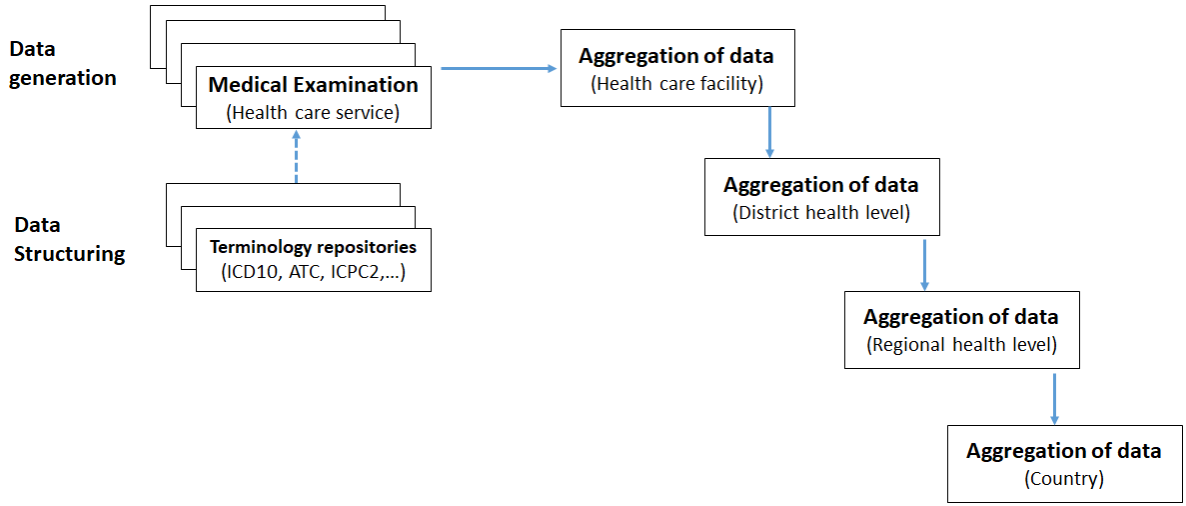
Production process of the source and group requirements graph. ATC: Anatomical Therapeutic Chemical; ICD: International Classification of Diseases; ICPC: International Classification of Primary Care.

Traditionally, when the system is not digitalized, all raw data are recorded on paper-based forms. Data processing at all levels becomes problematic. Indeed, at all decision-making levels, data are processed and aggregated manually (Figure 4). In this case, their semantic structuring, at least to give them a semantic coherence, might be done in a very subjective way. Manual matching is sometimes carried out subjectively by data managers or statisticians who proceed to perform the semantic matching of local medical concepts with other standard concepts of terminological reference systems such as the International Classification of Diseases (ICD)-10 for diagnoses and the Anatomical Therapeutic Chemical for drugs. The construction of the database model, on which the DMIS to be set up will be based, has, therefore, taken all these elements (as described in Figure 4) into account.

### Conceptual Modeling of the DMIS

The design of the DMIS considered two aspects: (1) the data production processes (bottom-up approach) and (2) the needs of the users (top-down approach). This led to the identification of 5 decision-making levels for the construction of the DMIS (Table 1).

**Figure 5:**
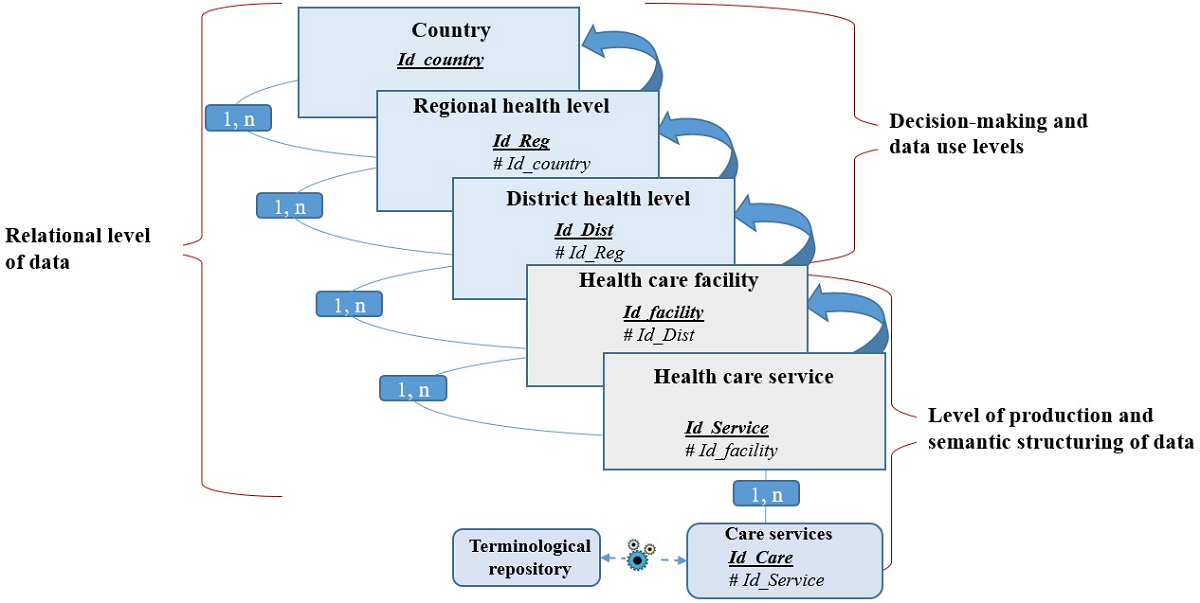
Conceptual data model of the proposed data warehouse.

Conceptually, this model is based on a relational database model (Figure 5). The objective is to propose a DMIS that integrates all the different data for the relational online analytical processing (ROLAP) solution to be performed to allow a multidimensional analysis of the data.

A digital interface was integrated into this relational database model, into which data managers enter data from the various hospital registers. This interface is simply a digital replica of the different hospital registers, using the same items, except for the data on patient identity. The data entered are detailed at the finest possible level of granularity and stored in the various relational tables of the tool. Then, a Structured Query Language (SQL) query was integrated to execute and generate a data table (a fact table) from which all multidimensional analyses can be performed.

The model summarized in Figure 5 integrates all the processes contributing to the performance of an HIS, from data collection to the dissemination of useful information for decision support. In this conceptual data model, each decision-making level was organized as a relational table. The tables were then linked together by logical relationships. These relationships made it possible to identify all the existing correlations among the data stored in different tables. This later facilitated their extraction and loading into a table generated using SQL queries.

The structuring of data before they are loaded into the DW has always been one of the major obstacles to the successful implementation of DMIS [18-21]. In organizations where the operational level is digitalized, this constraint is often solved by using an ETL tool that extracts and transforms the data before they are loaded into the DW. This allows a significant gain in data production time, with efficiency and precision in terms of data quality.

This study was set up in a context where most operational systems (care structures) did not have patient records digitalized, with terminological standards such as ICD-10 integrated to structure medical data and facilitate their automatic processing [22].

Often, to semantically standardize vocabulary terms, data managers or statisticians had no choice but to manually execute their correspondence with standardized terms of ICD-10, which not only made the task of collecting, processing, and disseminating data very laborious but also affected the quality of data in terms of completeness and delayed the production of statistics.

**Table 1:** Levels of data production and use of the produced data for decision-making.

Levels	Types of links between the different levels	Types of data production and use of the produced data for decision-making
Country	Composed of one or more health regions, but a health region belongs to only one country	Forth level of data aggregation. The country aggregates data from all health regions for national decision-making.
Regional health level	Composed of one or more health districts, but a health district belongs to only one health region	Third level of data aggregation. The health region aggregates data from all health districts for regional decision-making.
District health level	Composed of one or more health facilities, but a health facility belongs to only one health district	Second level of data aggregation. The health district aggregates data from all health care facilities for decision-making at the health district level.
Health care facility	Composed of one or more health care services, but a health care service belongs to only one health care facility	First level of data aggregation. The facility aggregates data from all health care services for decision-making at the facility level.
Health care service	Produces data during one or several medical consultations per day, but a medical consultation can occur at only one medical service	The health care service is the primary producer of data, as it provides various medical services including but not limited to drug prescriptions, medical examinations, and medical diagnoses on behalf of the medical facility to which it belongs. Therefore, the service is the producer of all health information in the health system.

To address this issue, an algorithm was defined to help the semantic matching of data and was integrated into a tool that enables the structuring of data and facilitates their automatic processing.

The proposed tool provides a graphical user interface, allowing the user to map an unstructured term with a structured term of ICD-10 from other platforms of assistance to coding or semantic annotation based on medical ontologies. This was done to match medical terms with each other and strengthen their understanding by gaining access to more detailed knowledge elements, such as definitions and synonyms [17]. A match score between 0 and 1 was automatically generated to evaluate the quality of a given match. This score was calculated from the total number of matches established between a given local term and a given standard term. The closer its value was to 1, the better the match.

**Figure 6:**
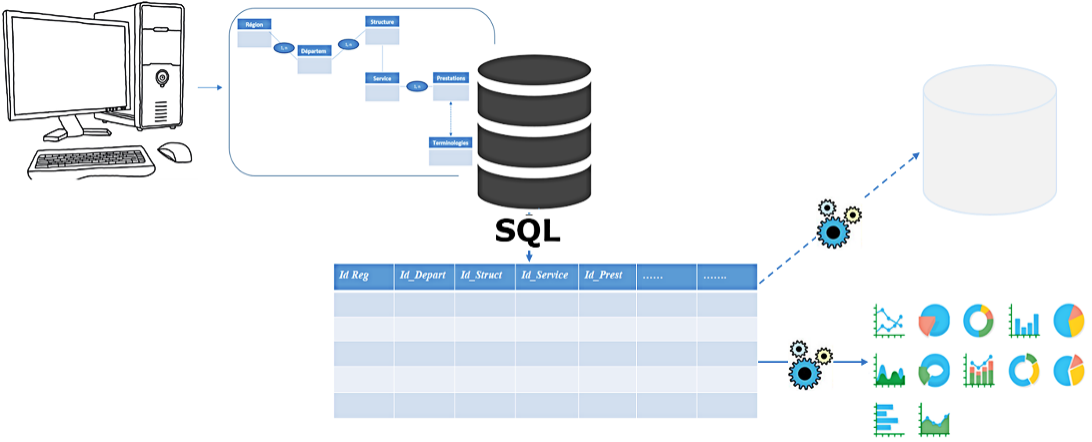
Architecture of the proposed platform model and SQL query generating the fact table. SQL: Structured Query Language.

The ROLAP approach was used for the multidimensional representation of data. The ROLAP, using an SQL query–generated table containing the results (primary keys and structured data of the different tables) (Figure 6), allowed all analyses to be performed from several decision angles.

**Figure 7:**
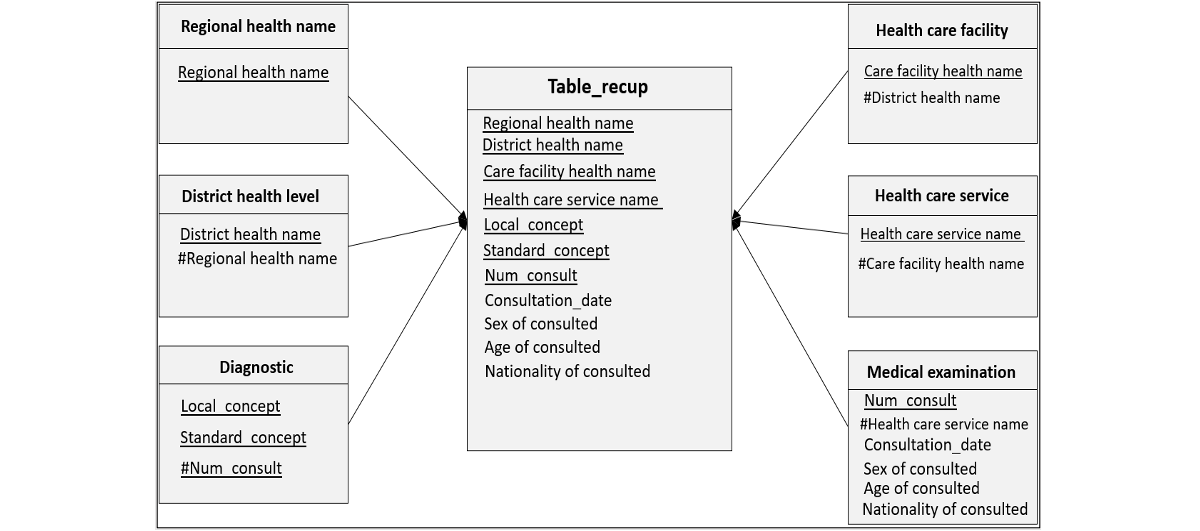
Example of a star diagram of the model centered on a join query.

Figure 6 describes the architecture of the platform implemented in Gabon as well as the SQL query that allows the raw data to be stored at the finest possible level of granularity in a materialized view, which is assimilated to a fact table and integrates the data contained in the various tables of the database deployed in the platform. This materialized view avoids manual processing (ETL) of data before integration into the DW. It uses the primary keys of all other tables as foreign keys to retrieve data from all the relational tables (Figure 7). Thus, a simple multidimensional star model containing the following dimensions was designed as shown in Figure 7.

Geographical location dimensions were as follows:

- Regional health level (regional health name)
- Health district level (health district name and regional health name)
- Health care facility (health care facility name, administrative category, and district health name)
- Health care service (health care service name and health care facility name)

Medico-administrative dimensions were as follows:

- Consultation (Num examination, examination date, patient sex, patient age, patient nationality, Consultation Residence, and Service ID)
- Diagnosis (CodeICD10 or ICPC2, local concept (diagnosis), standard concept (diagnosis), and Num examination)
- Drug (Anatomical Therapeutic Chemical code, local concept, standard concept, and Num examination)
- Laboratory (CodeLOINC, local concept, standard concept, and Num examination)

The table of facts contained the following:

- Retrieval table (Regional health name, health district name, health care facility name, health care service name, Num examination, CodeICD10 or ICPC2, CodeATC, CodeLOINC, Examination date, patient sex, patient age, patient nationality, patient habitation, local concept [diagnosis] standard concept [diagnosis], local concept [medicine], standard concept [medicine], local concept [biology], standard concept [biology], etc)

In contrast to the current HIS in Gabon, where data are aggregated manually, the model developed in this study allows data integration automation and the performance of relevant analyses and visualizations with more detailed levels of granularity. Using this model, there is a possibility to provide meaningful information from the data by considering the time such as day, week, month, and year, and the provenance of the data, including the health department, health region, and country [23].

### Design of the DMIS

The DW was implemented using the MySQL relational database management system with a multidimensional ROLAP data model.

The implemented system, referred to as *Routine Info*, has a general interface allowing the user to connect using a log-in and password attached to one of the decision levels (health service, health facility, health district, regional health, and country), which constitute, at the same time, different connection profiles to the tool with functionalities distributed as shown in Table 2.

**Table 2:** Accessibility level of functionalities according to profiles.

Functionality profiles	Create a record	Record clinical data	Match local concepts to standard concepts in terminology repositories	Update data entered (delete, modify, or add)	Analyze and perform multidimensional cross-checking of data	Visualize data	Exporting service data
Health care service profile	✓	✓	✓	✓	✓	✓	✓
Health care facility profile				✓	✓	✓	✓
District health profile				✓	✓	✓	✓
Regional health profile				✓	✓	✓	✓
Country profile				✓	✓	✓	✓

### Implementation of the DMIS Pilot

For the implementation and testing of the *Routine Info* platform, 2 health regions of Gabon were chosen. For 2 months, the platform was routinely used simultaneously in a health center, the regional hospital, and a university hospital to respond to the 3 levels of the health pyramid (department, region, and country).

The data managers of these facilities were asked to fill in the platform with the raw data available in the medical visit registers. The data filled in included clinical, demographic, and socioeconomic data. The objective was to compare the quality of the data reported using the Routine Info platform with that of the data produced by the traditional system used within the HIS in Gabon. During the 2 months of use, actual data from various medical consultations were integrated in near real time. For reporting purposes, Tableau 2020.1 software was connected to this database for data analysis and visualization.

The implementation of the platform in these 2 regions made it possible to obtain data associated with medical consultations recorded from January 20, 2018, to July 12, 2018. The results gathered made it possible to carry out multivariate analyses at all levels of the health pyramid. These results have been shown to support the decisions of decision makers at all levels.

### Scalability and Evaluation of the Pilot

To scale up the model, a pilot implementation phase was carried out over a period of 2 months in health facilities in 2 of the 10 health regions of Gabon. These 2 health regions serve 49% (8,87,428/1,811,079) of the general population, employ 34% (2125/6250) of health professionals, and have 31.95% (333/1042) of the health facilities in Gabon [13].

At the end of this pilot phase, the Routine Info tool was evaluated through a survey interview of all the users who participated in the pilot phase.

This evaluation was based on 3 determinants contributing to the achievement of data quality, namely the ability to have fully recorded data, the ability to facilitate the verification of data accuracy, and the ability to have all data available in a timely manner.

The evaluation method used was a comparative one, whereby the surveyed users filled in 2 different questionnaires with identical questions to evaluate and compare Routine Info with the traditional system. The questionnaires consisted of the following questions:

- Does the proposed system have a database that stores all data recorded in the facility daily?
- Does the proposed system allow for automatic data recording?
- Does the system allow for real-time data entry of all the data contained in the register?
- Does the system allow for the timely production of statistics?
- In how many days, on average, can the user have the statistics with the proposed system?
- With the proposed system, did the user produce the report for the previous month in a timely manner, meaning by the 5th of the current month?
- Is it possible to disaggregate the data by health facility and by service in the system?
- With the proposed system, is it possible to disaggregate the data and check its conformity with the data contained in the register of a given service?
- Is it possible to check the accuracy of the data with the proposed system?
- What percentage of completeness does the user think is achievable with the system?
- Is it possible to automatically aggregate the data of a given health service with the help of the system?
- How do you rate the process of processing National Health Information (NHI) data with the proposed system?

The collected data made it possible to carry out a qualitative analysis with the calculation of the frequencies and scores associated with these assessment criteria. Using the McNemar test, the aggregate data scores obtained for the 2 systems were compared.

### Ethics Approval

After approval was obtained from the ethics board of each hospital, all the persons participating in the evaluation were fully informed about the evaluation being conducted, and they explicitly agreed through a consent form. Further, they participated in the evaluation free from any coercion and were informed about their rights to be free to withdraw their participation at any time without negatively impacting their involvement in their respective hospital activities.

We provided an anonymous web-based form to be filled out for the evaluation and for further analysis. No personal or identification information was collected during this study. The participants gave their free and informed consent to the publication of the results of this study.

## Results

### Feedback on the Implementation of the Pilot and Evaluation

To test and evaluate the proposed system, a set of health professionals were first randomly selected from the participating health facilities in the 2 health regions of Gabon. Then, these professionals participated in various test sessions. Finally, we interviewed them to collect their opinions. In total, 14 health professionals (3 women and 11 men) were interviewed and participated in the various testing and evaluation sessions of the prototype. Overall, they had a mean age of 43 (SD 7) years and 12 (SD 1) years of experience in HIS data management. Specifically, there was no statistically significant difference between women and men in terms of age (*P*>.05) or experience in the NHI system (*P*>.05).

The 14 health professionals interviewed were distributed as follows: statistics specialist, 36% (5/14); data managers, 29% (4/14); activity coordinator, 14% (2/14); physician: 7% (1/14); epidemiologist: 7% (1/14); and computer scientist, 7% (1/14).

### Capabilities of the Tools to Produce Quality Data

**Figure 8:**
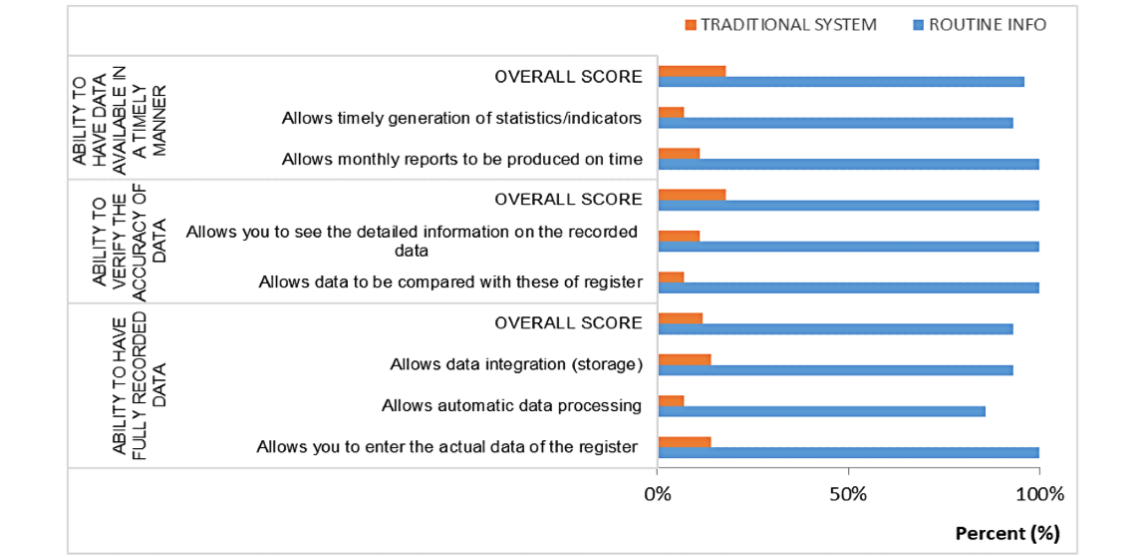
Comparative results on the ability of the tools to produce quality data from the aggregate data score.

Figure 8 shows a short description of the results derived from the analysis of the questionnaires about the capacity of the tools to produce quality data.

The ability to obtain all health data in a timely manner resulted in an overall response score of 96% (27/28) for all data quality criteria for the Routine Info tool compared with an overall response score of 18% (5/28) for all data quality criteria for the traditional system.

Overall, 2 criteria were used to assess the ability of the system to provide all data in a timely manner. These 2 criteria were the ability to produce monthly reports on time and the ability to generate statistics or indicators on time. These 2 criteria had scores of 93% (13/14) and 100% (14/14), respectively, for the Routine Info tool compared with 7% (1/14) and 28% (4/14), respectively, for the traditional system that met these criteria.

With regard to the ability to facilitate the verification of data accuracy, the Routine Info tool had an overall response score of 100% (28/28) for all data quality criteria compared with a score of 18% (5/28) for the traditional system.

The 2 criteria that were used to assess this dimension were the ability to compare the tool’s data with the registry data and the ability to have the registered data detailed at the finest possible level of granularity. These 2 criteria had scores of 100% (14/14) each for the Routine Info tool and 28% (4/14) and 7% (1/14), respectively, for the traditional system.

Regarding the ability to have fully recorded data, the results showed that the Routine Info tool had an overall response score of 93% (39/42) for all data quality criteria, compared with a score of 12% (5/42) for the traditional system.

A total of 3 criteria were used to assess this dimension. Measured individually, these criteria had scores of 100% (14/14), 86% (12/14), and 93% (13/14), respectively, for the Routine Info tool, compared with the scores of 14% (2/14), 7% (1/14), and 14% (2/14), respectively, for the traditional system.

The results presented in Table 3 show that according to the users surveyed, the quality of the data (completeness, accuracy, and timeliness) produced by the 2 systems was statistically different (*P*<.001). Routine Info had a higher capacity to produce quality data than the traditional system.

The respondents felt that the use of Routine Info allowed them to have access to data in a shorter period, which allowed them to make decisions quickly. They estimated that with Routine Info, it is possible to obtain data within an average of 1 day, whereas it takes an average of 21 days to obtain data with the traditional system. This feedback shows that the respondents agree that Routine Info significantly (*P*<.001) improves the time taken to provide data compared with the traditional system.

Following the test phase, 86% (12/14) of the respondents declared that Routine Info allows them to reach a completeness of more than 80%, contrary to the traditional system, where only 7% (1/14) of the respondents declared its capacity to allow the same completeness (Table 4).

**Figure 9:**
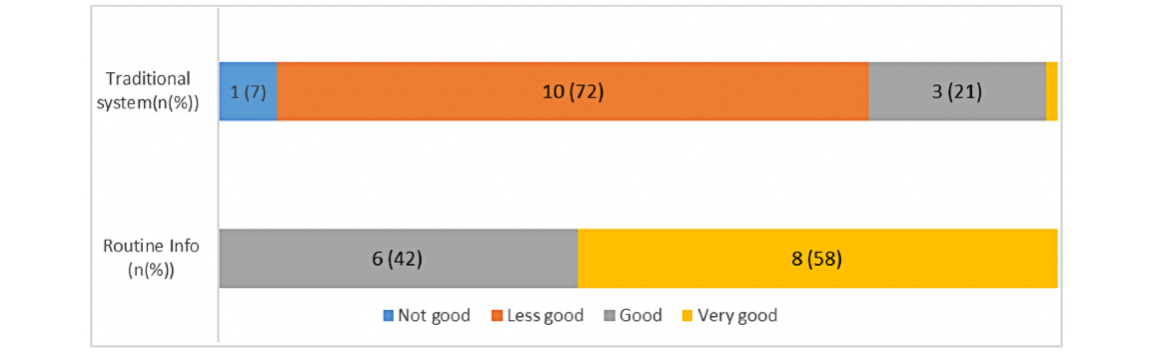
Satisfaction of users with the 2 tools.

Overall, the users were satisfied with Routine Info*,* as shown in Figure 9. Indeed, when asked, “How do you rate the NHI data processing process with the proposed system?” 43% (6/14) and 57% (8/14) of the users thought that the process was good and very good, respectively, with Routine Info. However, with the former traditional system, only 21% (3/14) of the users thought that the process was good, and 72% (10/14) declared it to be less good.

**Table 3:** Comparison between the capacities of the tools to produce quality data.

Score criteria	Routine info	Traditional system	*P* value
Score in relation to the ability to have completely recorded data	93	12	<.001
Data readiness score	96	18	<.001
Score in relation to the verification of the accuracy of the data	100	18	<.001

**Table 4:** Comparison of the completeness of the data estimated for each tool by the 14 users surveyed.

Estimated completeness achievable by the tool (%)	Traditional system, n (%)	Routine info, n (%)
<30	1 (7)	0 (0)
30-50	11 (78)	0 (0)
50-80	1 (7)	2 (14)
≥80	1 (7)	12 (86)

## Discussion

### Overview

The work reported in this paper illustrates a model and framework for improving the adequacy of information systems in low- and middle-income countries with limited digital infrastructure. A particular focus is placed on Gabon, where a pilot has been implemented and tested. The model proposed here constitutes the first initiative of this scale in the country. The inadequacy of the currently existing tools is often characterized by a low level of digitalization of the main data sources (operational systems). This leads to manual processing of health data. As a result, the quality is seriously affected to such a degree that the data are often incomplete, inconsistent, subject to long delays, and very often inaccurate. After testing and evaluating the proposed system in 2 regions representative of the 10 health regions in Gabon, feedback from the surveyed users showed that the proposed approach contributes to improving data quality and helps address the problems around data collection, data storage, and multidimensional data processing, processes which are often found to be not available in the health systems in developing countries [11]. Although various initiatives were conducted with international donors to improve the HISs and, in turn, the quality of data in these countries, these initiatives unfortunately lack sustainability after the donors withdrew. Indeed, the presence of a multitude of nonharmonized tools complicates the data collection process for local data managers. Thus, they have to collect all the data sent to them or available to them by manually filling in all the tools provided to them.

### Value of the Model Compared With Existing Models

The design and implementation of a system based on a relational database approach, which integrates all data production processes, is a real challenge, as it pertains to facilitating the collection and structuring of data available on paper registers. It helps to structure different data and make semantically coherent data recorded in the different tables of the system. Furthermore, it helps to meet another major challenge of improving data quality. This systematically excludes manual and subjective manipulation despite the absence of digitalized operational systems, as is the case in the context of an assertive digital transformation. Indeed, in a context of sufficient digital maturity, many platforms such as i2b2 [20] and Ehop [17] have simply used ETL tools to extract and transform data to the platform model before loading [16].

As an ETL tool cannot be used because the data are not digitized at the source level, the problem of centralizing the data is therefore taken into account by an SQL query that carries out the selection of the relevant health information in the various tables and makes them persistent in an extraction table. The latter is a materialized view of the data on which several analyses can be carried out later on. As the common model of the Observational Medical Outcomes Partnership platform represents the common structure of the data in the Observational Medical Outcomes Partnership on which all possible processing of the data is performed, the materialized view (considered as a retrieval table) resulting from the SQL query of Routine Info is, therefore, the main table on which all processing around the data will be done. However, the platform has the distinct advantage of integrating a natural language processing program to automatically structure the free-text data of electronic patient records [18].

### Benefit in Data Storage and Processing From the Proposed Model

It is in fact from the retrieval materialized view of the data that all the relevant data for decision support are centralized, thus allowing users to dynamically generate various statistics. This model, which records the data from the department in which it is produced, now excludes manual processing to fill in the multiple tools that make the work of data managers repetitive and laborious. Furthermore, the verification of the accuracy of the data by comparing the observations of the reports and registers is therefore taken into account upstream. This makes it easier to assess the quality of data, for example, with the Performance of Routine Information System Management approach [3].

The implemented system makes it possible to disaggregate data from the country level down to the service level and assess whether the data contained therein really reflect those present in the production sources (eg, consultation registers). In a context of insufficient digital infrastructure at the level of the operating systems with, for example, nondigitalized HISs, as can be observed in several information systems [18], this model responds well to the question of automatic extraction and integration of data by avoiding manual processing (and preprocessing) of data. Indeed, because of the nonexistence of digitized transactional databases, an initial processing consisting of counting the data by type of diagnosis and type of drug prescription is often carried out by staff members before filling in the aggregated totals in DWs. This approach, which our present framework avoids, did not allow for the most detailed analyses. This is because the data aggregated manually and entered into the DW are not related to their primary sources, which makes it very difficult to check the accuracy or completeness of the data, for example.

Therefore, this proposed model and framework has the advantage of acting as a transactional database for data integration into other platforms. This is specifically the case of HISs in many developing countries that use the DHIS2 platform [10]. Indeed, the DHIS2 platform is often populated at the district level from preprocessed, manually aggregated data, whereas for more detailed and better quality data [4], it is necessary to integrate the health structure level and, when existing, the health service level, which are both already taken into account in our model. In terms of the fragmentation of information systems, which multiplies the collecting tools for health structures, this study provides a solution in that a data manager will only have to query the database to fill in the various collecting tools.

### Limitations

A scale-up was carried out only in 2 pilot health regions, while reducing the size of the sample of interviewees; this could constitute a limitation in the exploitation of the results of this work. A countrywide or subregional implementation is envisaged to continue the testing and validation process of the proposed model.

Another limitation is the fact that only the general admission service with routine consultations and diagnostics is taken into account. It is worth implementing the framework in various units of hospitals, including those of intensive care.

### Conclusions

The proposed model and framework integrates all the processes of data collection, processing, and dissemination, thus providing complete, accurate, and near real-time data availability. Indeed, because data collection is mainly done at the level of the service in which these data are produced and the data are used at all levels for decision-making, our approach contributes significantly to ensuring the improvement of data quality for the management of health systems in the context of limited infrastructure. In addition, the approach also allows for multidimensional analysis and the provision of dashboards necessary for monitoring and evaluating health program indicators, both at the national and local levels.

## Abbreviations

DHIS2: District Health Information Software 2
DMIS: decision-making information system
DW: data warehouse
ETL: extraction, transformation, and loading
HIS: health information system
ICD: International Classification of Diseases
NHI: National Health Information
ROLAP: relational online analytical processing
SQL: Structured Query Language
SSA: Sub-Saharan African
